# Pan-cancer integrative analysis of whole-genome De novo somatic point mutations reveals 17 cancer types

**DOI:** 10.1186/s12859-022-04840-6

**Published:** 2022-07-25

**Authors:** Amin Ghareyazi, Amirreza Kazemi, Kimia Hamidieh, Hamed Dashti, Maedeh Sadat Tahaei, Hamid R. Rabiee, Hamid Alinejad-Rokny, Iman Dehzangi

**Affiliations:** 1 Bioinformatics and Computational Biology Lab, Department of Computer Engineering, Sharif University of Technology, Tehran, 11365 Iran; 2 Department of Computer Engineering, Simon Fraser University, Burnaby, BC 1S6 Canada; 3 Department of Computer Science, University of Toronto, Toronto, ON M5S 3H2 Canada; 4 BioMedical Machine Learning Lab (BML), The Graduate School of Biomedical Engineering, UNSW Sydney, Sydney, NSW 2052 Australia; 5 UNSW Data Science Hub, The University of New South Wales (UNSW Sydney), Sydney, NSW 2052 Australia; 6 AI-Enabled Processes (AIP) Research Centre, Macquarie University, Sydney, 2109 Australia; 7 Department of Computer Science, Rutgers University, Camden, NJ 08102 USA; 8 Center for Computational and Integrative Biology, Rutgers University, Camden, NJ 08102 USA

**Keywords:** Pan-cancer, Somatic point mutations, Cancer subtyping, Biomarker discovery, Driver genes, Personalized medicine, Health data analytics

## Abstract

**Background:**

The advent of high throughput sequencing has enabled researchers to systematically evaluate the genetic variations in cancer, identifying many cancer-associated genes. Although cancers in the same tissue are widely categorized in the same group, they demonstrate many differences concerning their mutational profiles. Hence, there is no definitive treatment for most cancer types. This reveals the importance of developing new pipelines to identify cancer-associated genes accurately and re-classify patients with similar mutational profiles. Classification of cancer patients with similar mutational profiles may help discover subtypes of cancer patients who might benefit from specific treatment types.

**Results:**

In this study, we propose a new machine learning pipeline to identify protein-coding genes mutated in many samples to identify cancer subtypes. We apply our pipeline to 12,270 samples collected from the international cancer genome consortium, covering 19 cancer types. As a result, we identify 17 different cancer subtypes. Comprehensive phenotypic and genotypic analysis indicates distinguishable properties, including unique cancer-related signaling pathways.

**Conclusions:**

This new subtyping approach offers a novel opportunity for cancer drug development based on the mutational profile of patients. Additionally, we analyze the mutational signatures for samples in each subtype, which provides important insight into their active molecular mechanisms. Some of the pathways we identified in most subtypes, including the cell cycle and the Axon guidance pathways, are frequently observed in cancer disease. Interestingly**,** we also identified several mutated genes and different rates of mutation in multiple cancer subtypes. In addition, our study on “gene-motif” suggests the importance of considering both the context of the mutations and mutational processes in identifying cancer-associated genes. The source codes for our proposed clustering pipeline and analysis are publicly available at: https://github.com/bcb-sut/Pan-Cancer.

**Supplementary Information:**

The online version contains supplementary material available at 10.1186/s12859-022-04840-6.

## Introduction

Cancer is a heterogeneous disease characterized by the progression of molecular changes that can develop in different tissues. Many tumors within a tissue have different molecular mechanisms. Moreover, some tumors across multiple tissues appeared to have similar biological mechanisms [1–4]. Different histology, mutation profiles, or expression profiles can distinguish tumors into several subtypes, enabling us to classify patients into subgroups with similar clinical characteristics or medical diagnoses better than cancer types. Pan-cancer classification is a relatively new approach aiming to understand the origin and cause of all cancer types. Although cancer types have many differences, we consider them a single disease. Hence, by subtyping this disease, we can better understand its origins and causes. New studies in this field provide us with promising findings [2].

During the last few years, cancer subtype identification has been performed using expression data [5, 6], copy number [7], DNA methylation data [8, 9], or integration of different omics types [10, 11]. For instance, [12] employed three different similarity kernels on three types of profile data (gene expression, miRNA expression, and isoform expression data) for five cancer types from TCGA and then aggregated computed similarities by using the Similarity Kernel Fusion (SKF) for tumor subtyping. In [10], the authors used a hierarchically stacked autoencoder (called HI-SAE) on the gene expression and transcriptome alternative splicing profiles to learn new data representations. Then, based on the newly learned data representations, they classified breast cancer patients from TCGA.

Transcriptional profiling of samples has multiple issues, including the effect of invasive sampling and its impact on expression profiles and noise in collected data. At the same time, mutational profiles are more robust to these problems [13]. However, some studies have tried to perform identification based on the somatic point mutations instead of the expression data. Somatic mutation is closely related to cancer due to its essential role in cancer progression. Since mutational processes or genes involved can be linked to different molecular mechanisms driving tumor progression and clustering tumors based on this data type can be very informative and effective. However, existing sparseness (many samples have only a limited number of mutations) and heterogeneity (two tumors rarely share the same mutations) in mutation data bring new challenges.

Some studies have addressed the sparseness issue by using gene interaction networks as prior knowledge. For instance, [14] applied an algorithm called NetNorM to raw somatic mutation data and constructed more amenable data by employing Pathway Commons (a dataset containing gene network information). They removed non-essential mutations for high-mutated tumors to creating normalized data and added missing mutations for less-mutated tumors. [15] also used different gene interaction networks to construct smoothed mutational data by propagating driver mutated genes into their neighborhood in the genes network. This approach identifies sub-networks around a highly connected or mutated gene. Other studies, such as [16], proposed modifying heat (gene score) in the network to reduce the diffusion of genes like *TP53.* It was discussed in [17, 18]. that abnormality in gene expression or regulation rather than mutation causes some cancers [17, 18]. Even though gene expression and gene interaction networks can provide insight into the causes of some cancers, they cannot be used in all studies to uncover significant genes. This is particularly true in studies that focus only on mutational profiles. Moreover, it ignores possible indirect interactions between genes not captured in gene networks.

More recently, [19] resolved the data sparseness challenge by developing a de-sparsification method that summarizes somatic mutations in genes into pathway-level mutation scores. Then, they used the binomial distance to cluster pathway mutation scores. Although this method helps identify the mutational patterns associated with clinical phenotypes, they just focused on the previously cancer-associated genes [20, 21] to find pathway scores. As a result, this method is not the most suitable approach for cancer subtyping because already known genes may not fit the best model for mutational profiles. In other words, it does not consider the essential unknown genes that might play a significant role in developing cancer. This study addresses this issue by finding the best-fitted distributions for each cancer type’s mutational profiles, which enabled us to identify the significantly mutated genes in each cancer by defining a threshold. We believe our approach can identify biologically important genes beyond using a set of previously identified cancer-associated genes for more accurate subtyping. In this way, pan-cancer subtyping using mutational profiles can become more precise, and mutational subtypes among cancer types can be better identified since more tumors are being examined.

To the best of our knowledge, mutational processes do not have the same effect on genes. This has never been adequately explored for cancer subtype identification in previous studies. By studying mutation rate among samples and mutational signatures in subtypes, we demonstrate that mutational processes do not have the same effect in different cancer types. While in our proposed classes of cancers, we show that this effect is homogenous among samples. This provides better insight for researchers and clinicians to understand the origin of a patient’s cancer and develop new treatments.

In this study, based on the idea of pan-cancer and the advantages of somatic mutations, we studied mutational profiles available from International Cancer Genome Consortium (ICGC), which contains tumors from 19 cancer types. We explored a wide range of statistical distributions for each cancer type to model mutational profiles and identify significantly mutated genes for each cancer type. Then, we performed a hierarchical clustering model on somatic mutations in these genes by aggregating identified candidate genes of each cancer type. Our clustering approach is based on the Gaussian Mixture Model (GMM), which outperforms other techniques such as K-means in similar studies. This method chooses the best number of clusters by evaluating various metrics. We started by performing model-based clustering on all tumors and iteratively repeated this process on the generated subgroups. No new meaningful subgroup was generated concerning the clustering threshold. As a result, we identified 17 subtypes. To investigate the effectiveness of our proposed subtyping approach, we provide a comprehensive analysis, including mutational load, gene association, mutational signature, gene ontology, pathway enrichment, and survival analysis for each subtype. These experiments help us indicate different distinguishable molecular mechanisms in each identified subtype. The source codes for our proposed clustering pipeline and analysis are publicly available at: https://github.com/bcb-sut/Pan-Cancer.

## Results and discussion

We performed a background model to extract significant coding genes to be able to distinguish cancer patients. Single-base mutational profiles of samples were obtained from the International Cancer Genome Consortium (ICGC) dataset. Using model-based clustering, we clustered 12,270 samples across 19 cancer types into new subtypes by considering extracted genes. Finally, we performed comprehensive biological analyses on our identified subtypes to investigate each subtype’s biological characterization and gain new insights into cancer subtyping.

### Pre-analysis

This study focuses on somatic point mutations from the ICGC dataset, containing 19 tissue cancer data. We used 12,270 cancer samples collected across different projects, including READ-US, COAD-US, COCA-CN, etc. We used the Ensemble gene annotation dataset [22] to identify several somatic point mutations in coding genes. This dataset contains 20,345 protein-coding genes. We excluded all non-single-base mutations (e.g., insertions, deletions) from our analyses. To identify new subtypes, we only considered coding mutations.

### Identifying candidate genes

**Fig. 1:**
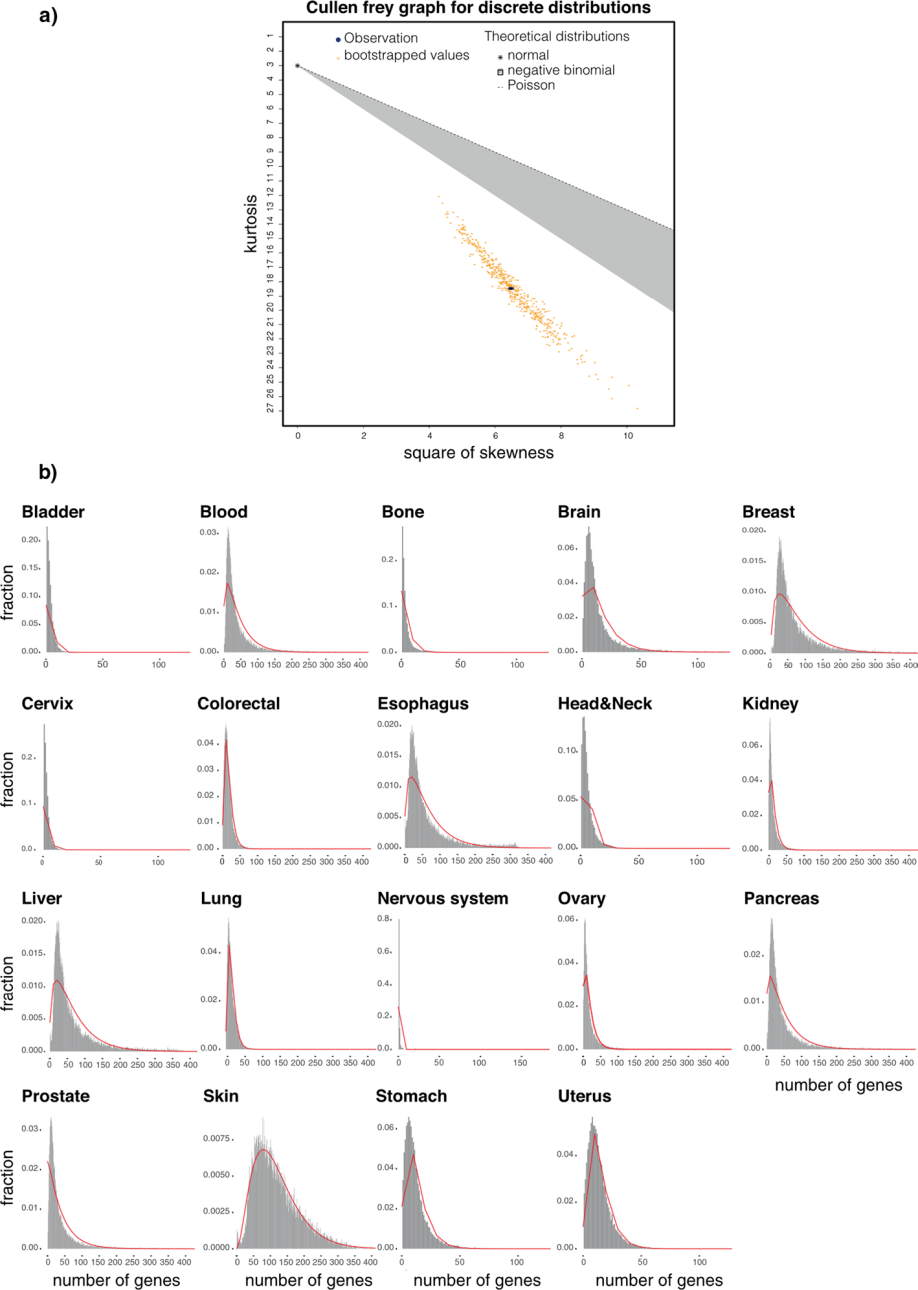
**a** Best-fitted distribution to discover feature genes. Cullen Frey method was applied to identify the best distribution fitting for mutational data of cancer types. The figure shows the Cullen-Frey graph for discrete distributions. **b** The distribution of mutated genes in cancer samples. We fitted a negative binomial to their mutational data for all cancer types. Each plot shows empirical mutation data in a specific cancer type, and the red line shows a negative binomial distribution fitted to the cancer type. The X-axis indicates the number of mutated genes, and Y-axis shows the fraction of samples in the specific cancer type. If a bar in x = 120 has y = 0.02, then there are 120 genes with mutations in 0.02 of samples of that cancer type. The distribution’s right tail points to mutated genes in more samples, therefore, more important genes

To cluster samples based on their mutational profiles, we first need to identify candidate genes that significantly mutated in each cancer. We first determined the best-fitted distribution to identify significantly mutated genes in each tumor. We used Cullen and Frey’s graph (*see method*) with 500-fold bootstrapping to find the best-fitted distribution (Fig. 1a). Although we examined various distributions, a negative binomial was the best-fitted distribution for all cancer types (Fig. 1b). Next, we used the Cramer-Von Mises test to confirm the perceived distributions. We considered each cancer type’s perceived distribution to detect candidate genes and then calculated the mutational load’s *p*-value for each gene. We then used a threshold of 0.001 on the *p*-value to determine candidate genes of each cancer type. We then gathered all candidate genes from all cancer types and identified 684 genes significantly mutated in at least one cancer. A complete list of candidates (features) genes and their *p*-value is provided (Additional file 1: Table S1).

The mutational load of feature genes in each cancer type is shown in (Additional file 2: Figure S2). According to this figure, some genes are significantly mutated in multiple cancers, which aligns with the idea presented by pan-cancer research. For instance, *TP53* and *KRAS* are examples of genes among the significant genes of many cancers such as Breast, Brain, and Ovarian. TP53 single-base substitution, the primary type of alteration, is associated with cellular proteins’ inactivation and leads to many cancers [23]. As the figure shows, pancreatic and prostate cancers are the most mutated cancers in the candidate genes (43.8% and 44.3% of the candidate genes are mutated in pancreatic and prostate cancers, respectively). Esophagus and nervous system cancers have fewer mutations among the candidate genes (only 1 and 2 genes out of 684 candidate genes are mutated in the esophagus and nervous system cancers, respectively).

### Model-based clustering to detect new subtypes

Having significantly mutated genes identified, we then used these genes as the features for our multi-level clustering approach to identifying cancer subtypes. For this purpose, we used the Mclust package implemented in R (see method section). We preferred this method over other clustering approaches because it builds a robust model to identify clusters without random initialization. Unlike Mclust, classic clustering algorithms such as k-means need to be randomly initialized and sensitive to initialization. Besides, clustering methods such as dbscan [24] and hdbscan [25] require the user to specify the optimal number of clusters. Hence, we applied the process based on Gaussian mixture models to overcome these issues and cluster our samples based on their inherent.

### Clustering method

**Fig. 2:**
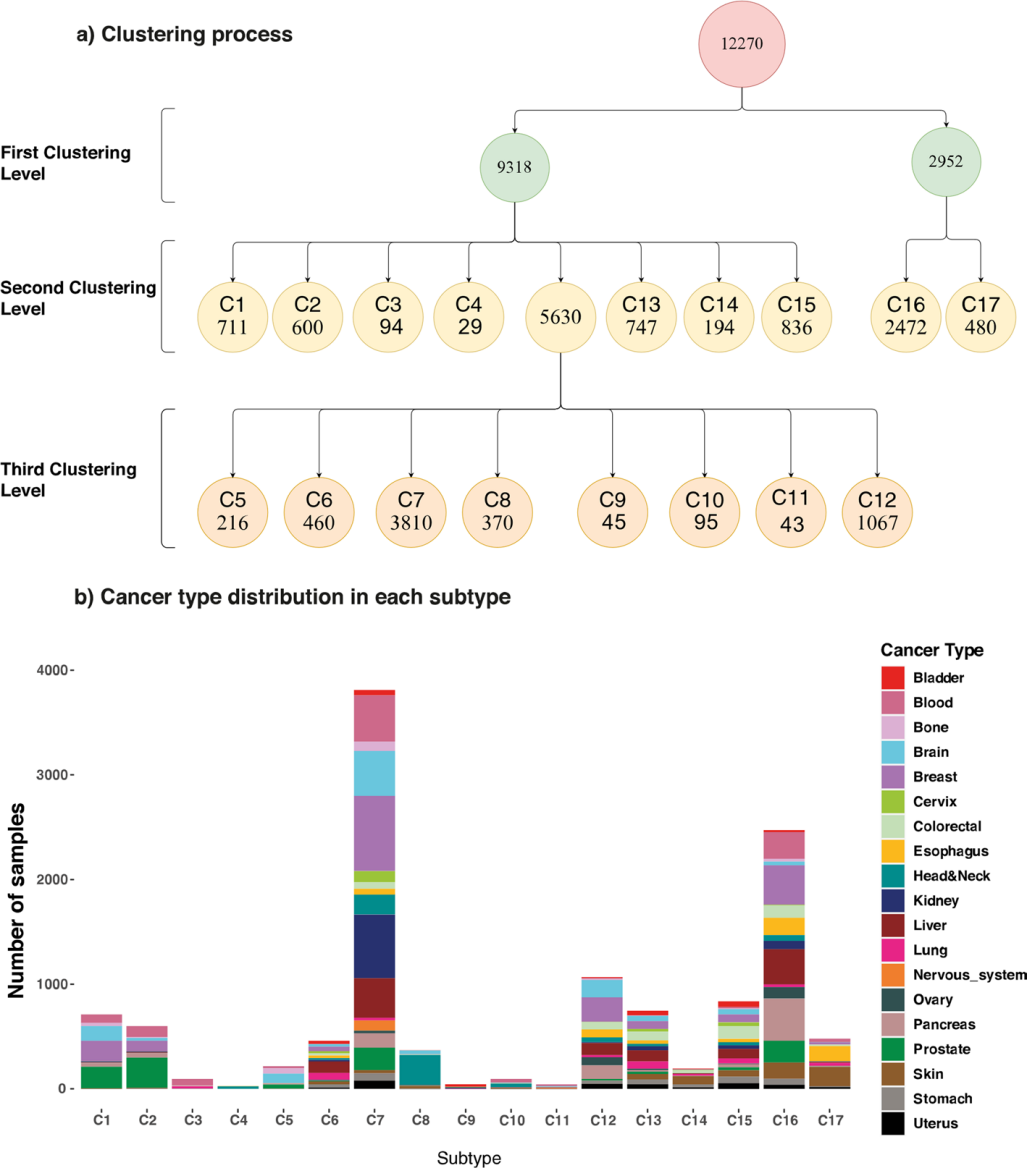
**a** The clustering tree shows the process performed by the Model-based method. In the first level of clustering, all 12,270 samples were divided into two sub-groups. In the second round of clustering, the first sub-group with 9318 samples was divided into eight sub-groups (1.1–1.8), and the second sub-group was split into two new sub-groups (2.1 and 2.2). And finally, the third level of clustering sub-group 1.5, with 5630 samples, was divided into eight sub-groups (1.5.1–1.5.8). **b** Distribution of all samples in identified subtypes. Each color corresponds to a cancer type. The X-axis shows subtypes, and Y-axis indicates the number of samples. Subtype C7 is the most populated subtype and comprises many samples from all cancer types (Kidney and Breast are observed in the C7 subtype more than other cancer types). Subtype C16, the next most populated, comprises samples from all cancer types (Pancreas, Liver, and Breast cancers are observed in C16 more than other cancer types)

We used model-based clustering to identify subtypes. Since we did not consider any assumption on several subtypes, we preferred a non-parametric method. Model-based clustering is one of the density-based and non-parametric unsupervised machine learning methods for clustering. Another reason to apply model-based clustering was sample independence and its number of mutations. Hence, we anticipated that candidate genes’ mutational load follows Gaussian distribution due to the central limit theorem if subtypes are precisely identified. Mclust is an available package in R, which we used to apply model-based clustering. Mclust fits each cluster's best Gaussian Mixture models and utilizes the Bayesian Information Criterion (BIC) metric to find the optimal number of clusters each time applied [26, 27]. Here, we hierarchically used Mclust with three levels of clustering. As shown in Fig. 2a, 12,270 samples are clustered into two clusters with 9318 and 2952 samples in each as it demonstrates the best clustering outcome for BIC. After that, each of these two clusters was given to the clustering algorithms. The results are illustrated in the second level of clustering in Fig. 2a. This process continued until no new meaningful subgroup was found or the algorithm returned a big cluster with more than 95% samples of the parent cluster and the rest to some small residual clusters.

As shown in (Fig. 2a), at the first level of clustering, the algorithm breaks down all samples into 2 clusters (Cluster 1 with 9318 and Cluster 2 with 2952 samples, respectively). Cluster1 was divided into eight sub-clusters (from Cluster 1.1 to Cluster 1.8), and Cluster 2 was split into two sub-clusters (Cluster 2.1 and Cluster 2.2). Finally, only Cluster 1.5 was divided into eight sub-clusters at the third level. The cutoff for new clusters was set at 95% as a conservative criterion for opting-out residual clusters (having less than 0.05 dropout). When a cluster was divided into multiple sub-clusters in which at least one contains more than 95% of the parent’s samples, we infer that all sub-clusters could be outliers, so the cluster should not be divided. As a result, we obtained 17 clusters as our new identified subtypes (17 subtypes from C1 to C17). Additional file 1: Table S2 shows all samples identified in each subtype.

We then investigated the contribution of samples from different cancers in our identified subtypes. Figure 2b shows this contribution as a bar plot, and Additional file 2: Figure S3 shows the contribution as a heat map. The number of contributing samples concerning different cancers in our identified subtypes is also provided in Additional file 1: Table S3. As we can see in both figures, most subtypes consist of various cancer types. For example, subtype C7 and subtype C12 contain all cancer types, but subtype C4 and subtype C8 are mainly composed of Head & Neck Cancer with 78% and 82% of their samples.

Most subtypes contain samples from 2 to 4 cancer types. For instance, Subtype C2 mainly consists of Prostate (48.2%), Blood (17.5%), and Breast (16.8%) cancers which are about 82.5% of all samples in this subtype. Subtype C3 consists of Blood (68.1%) and Lung (24.5%) types which are about 92% of all samples in this subtype, and subtype C9 also contains samples from Bladder (53.3%), Kidney (26.7%), and Cervix (11.1%) types which are about 91% of samples. Moreover, many cancers are primarily scattered in 3 or 4 subtypes. For instance, more than 95% of the Prostate cancer samples are grouped in C1 (19.8), C2 (27.9%), C7 (20.6%), and C16 (20%). The esophagus samples are primarily in C16 (26.7%) and C17 (30.6%), and more than 75% of Ovary samples are in C16 (44.9%) and C12 (31.4%).

In this study, similar to prior studies in pan-cancer and cancer subtype identification, we determined subtypes using unsupervised learning methods. In pan-cancer analysis, the aim is to examine the similarities and differences among the genomic and cellular alterations found in different cancers. The validity of clusters in unsupervised methods can only be determined by how they represent the data and similarity patterns in each cluster, mathematical analysis, and visualization. In fact, there is no definitive answer to the number of clusters and characteristics of subtypes due to the nature of the subtyping problem. In the following sections, we provide a comprehensive analysis, including mutational load, gene association, mutational signature, gene ontology, pathway enrichment, and survival analysis for each subtype to demonstrate the biological characterization of identified subtypes and show the effectiveness of our new subtyping approach over traditional cancer type classification approaches. These experiments help us indicate different distinguishable molecular mechanisms in each identified subtype. In this way, we investigate the validity of our proposed subtypes.

### Mutational load of genes for each subtype

**Fig. 3:**
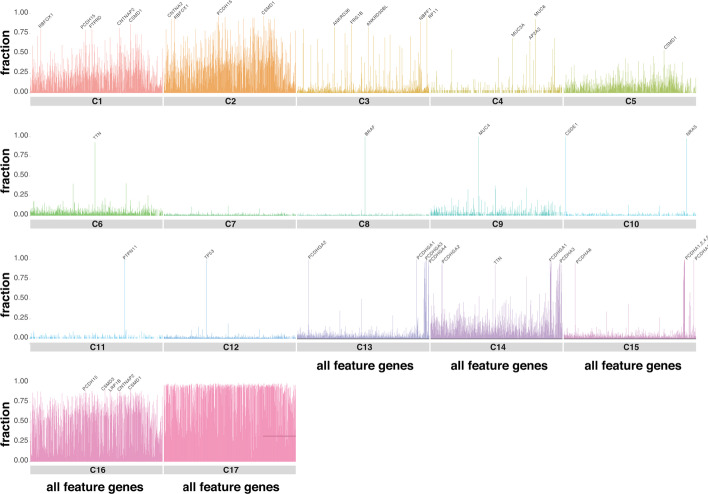
Each graph for each subtype illustrates the portion of samples in a subtype that has a mutation in each of the 684 genes. In other words, each bar indicates the number of samples that have a mutation in a gene among 684 genes, divided by the total number of samples in that subtype. The taller a bar, the more important that gene is for that subtype because more samples had a mutation in that specific gene

This analysis studies the mutational load of candidate genes and all protein-coding genes in our subtypes. To compute the mutational load of gene ‘g’ in subtype ‘C,’ we counted the number of samples in subtype ‘C’ which have a mutation in gene ‘g’ and then normalized it (by dividing it into the number of all samples of subtype ‘C’). As shown in Fig. 3, the distribution of mutational load of candidate genes in the identified subtypes is different. As the shown in this figure, subtypes C16 and C17 are hyper-mutated subtypes (5 genes with at least one mutation in 90% of samples in C16 and 276 genes with at least one mutation in 95% of samples in C17) which can be a reason that samples of these two subtypes were separated from others at the first level of clustering. Notably, 92.6% of samples in C6 have a mutation in *TTN*; all samples in C8 have a mutation in *BRAF*; all C9 samples have a mutation in *MUC4*; 100% and 97.9% of samples in C10 have a mutation in *CSDE1* and *NRAS,* respectively; all samples in C11 have a mutation in *PTPN11*; all samples in C12 have a mutation in *TP53*; all samples in C13 have a mutation in *PCDHGA1*, *PCDHGA2,* and *PCDHGA3*. Moreover, 98.6%, 96.2%, 99.1 and 98.1% of samples in this subtype have a mutation in *PCDHGA4*, *PCDHGA5*, *PCDHGB1,* and *PCDHGB2,* respectively (gamma Protocaderins family highly mutated in this subtype); 96.9% of samples in C14 have a mutation in PCDHGA2,3,4 and PCDHA1,2,3. Moreover, 97.4% of samples in this subtype have a mutation in *PCDHGA1*; 99.7% of samples in C15 have a mutation in PCDHA1,2,3. In addition, 99.7%, 96.1%, and 98.8% of samples in this subtype have a mutation in *PCDHA4*, *PCDHA5*, and *PCDHA6,* respectively (alpha Protocadherins family highly mutated in this subtype).

Interestingly, we identify similar patterns for some of the subtypes. For instance, *CSMD1* and *RBFOX1* are highly mutated in both subtypes C1 and C2 (*CSMD1* and *RBFOX1* are mutated in 80.5% and 87% of samples in C1, respectively, and 95.1% and 96.8% of samples in C2, respectively). Other examples are *PCDHGA1* and *PCDHGA2,* which are mutated in almost all samples of C13 and C14. To understand the difference between similar subtypes (C1 and C2; C13 and C14), we plotted the fraction of samples that have at least three mutations in each candidate (feature) gene (Additional file 2: Figure S4). This figure shows that tumor samples in C2 and C14 have higher mutations than subtypes C1 and C13, respectively. In addition, we observed that *CSMD1* is mutated in 74% of the samples in C2. In comparison, only 42% of samples in C1 are mutated within this gene, meaning that the difference between C1 and C2 originated from the different mutation rates in significant common genes. Additional file 2: Figure S4 also shows *PCDHGA1* mutated in 6% and 43% of C13 and C14 subtypes, respectively, demonstrating the effect of mutation numbers in distinguishing these two subtypes. Another example for C13 and C14 is *PCDHGA2* which mutated in 5% and 37% of samples in C13 and C14. Results demonstrate that common genes have more mutations in C14 than C13.

### Gene and gene-motif association as a biomarker of each subtype

**Fig. 4:**
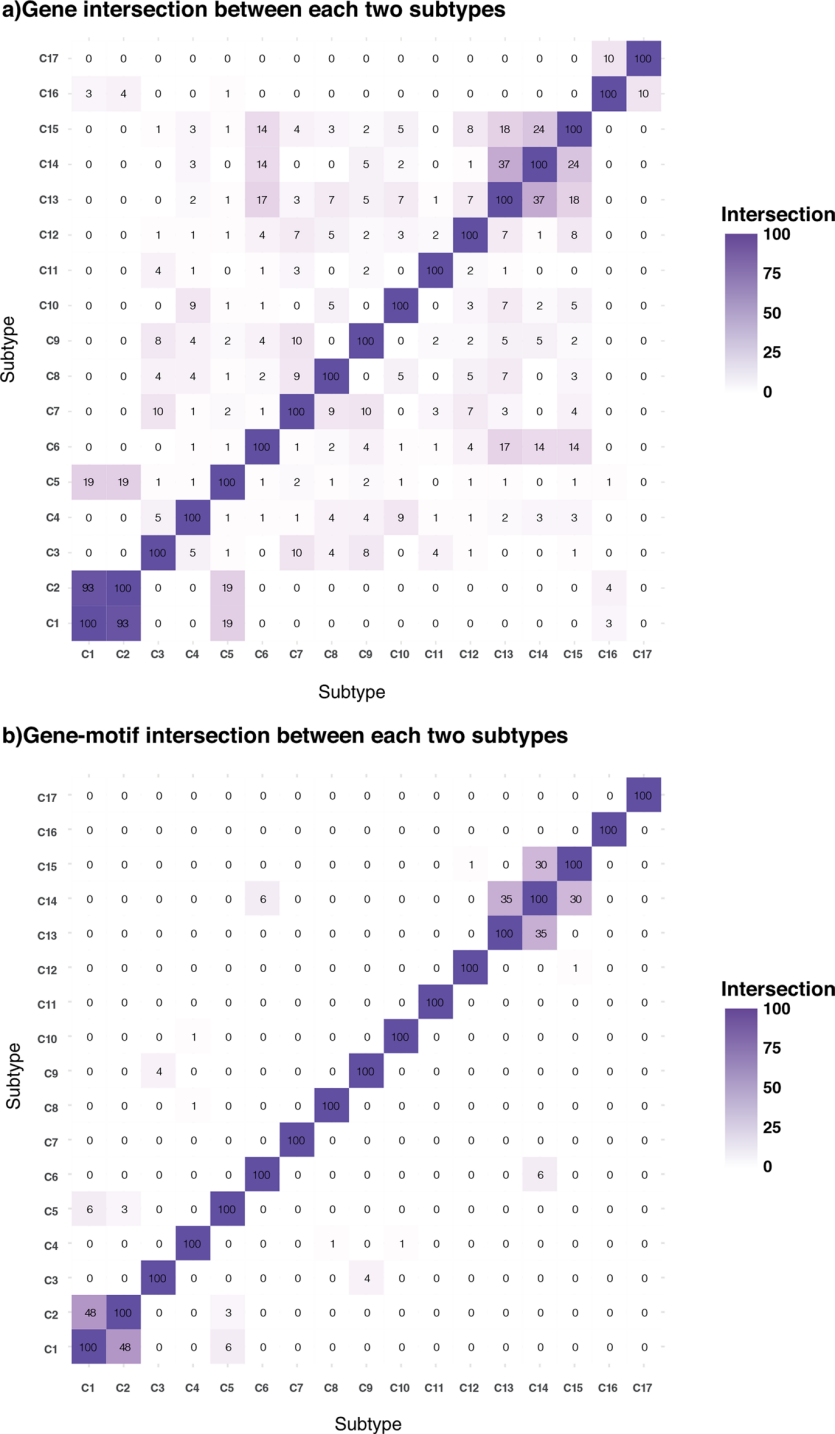
**a** Each cell corresponds to the number of genes in the top 100 significant genes among two subtypes. Many subtypes have very few genes in common with others except C1 and C2, 93 significant common genes out of 100, or C13 and C14, 37 significant common genes out of 100, or C13 and C15, have 24 significant common genes of 100. **b** Each cell corresponds to the number of gene-motifs in the top100 significant gene-motifs of every two subtypes. Interestingly to Fig. 4a, almost all subtypes have a fewer gene-motif in common than genes. For example, in subtypes C1 and C2, which have 93 genes in common, only 48 gene-motifs are in common. The only exception is C14 and C15, which have 30 gene-motifs in common, while these two subtypes also have 24 genes in common

We then investigated the top 100 highly mutated genes in each subtype (Additional file 1: Table S4) and asked how many of the top 100 highly mutated genes are common between every two subtypes. As shown in Fig. 4a, many pairs of subtypes have a few common genes, while others have numerous common genes. For example, subtypes C1 and C2 have 93 significant common genes out of 100 in both subtypes. While subtypes C13 and C14 have 34 common genes in their top 100 significant genes. Interestingly, there is no common gene between the top 100 important genes for many subtypes.

It has been recently shown in [28] that gene-motifs are the primary source of disease-related variations in cancer. Gene-motifs refer to the 3-nucleotide sequence mutated within a gene, i.e., NXN-to-NYN (where reference nucleotide X mutated to Y, and N: A, C, G, or T). There are 96 combinations of mutations within 3-nucleotide motifs. For example, *MUC16, LRRC4C,* and *IL1RAPL1* are examples of genes that appeared as significant genes within the top 100 important genes of different subtypes. We investigated each subtype’s mutations in tri-nucleotide motifs to show the motif preferences of mutations in each gene (Additional file 2: Figure S5).

Interestingly, our result (Additional file 2: Figure S5a) indicates that *IL1RAPL1* has more T > A and T > C mutations in C2 compared to C1 and C5 samples. In addition, among samples in C5, *IL1RAPL1* has mutations in a smaller number of motifs compared to C1 and C2. According to Additional file 2: figures S5b and 5c, the same results are observed for *LRRC4C* and *MUC16*.

Many shared genes between different pairs of subtypes (e.g., C1 and C2) led us to investigate the mutational loads within 3-mer motifs in the top 100 important genes, separately. We used Fisher exact test (*method section*) and individually identified significantly mutated motifs within the top 100 significant genes in each subtype. Considering the top 100 gene-motifs for each subtype, we identified common gene-motifs between every two subtypes, shown in Fig. 4b. Interestingly, this analysis more clearly shows the difference between the identified subtypes. Compared to Fig. 4a all pairs have less common significant gene-motifs than significant common genes (except C14 and C15, which have 30 common gene-motifs). There is no common gene-motif between most paired subtypes (Fig. 4b). Importantly, subtypes C1 and C2, with 93 significant common genes within their top 100 most mutated genes, have only 48 common gene-motifs (within their top 100 gene-motifs), showing different molecular mechanisms within these subtypes. The complete lists of the top 100 significant gene-motifs for each subtype are provided in Additional file 1: Table S5.

### Mutational signature analysis

**Fig. 5:**
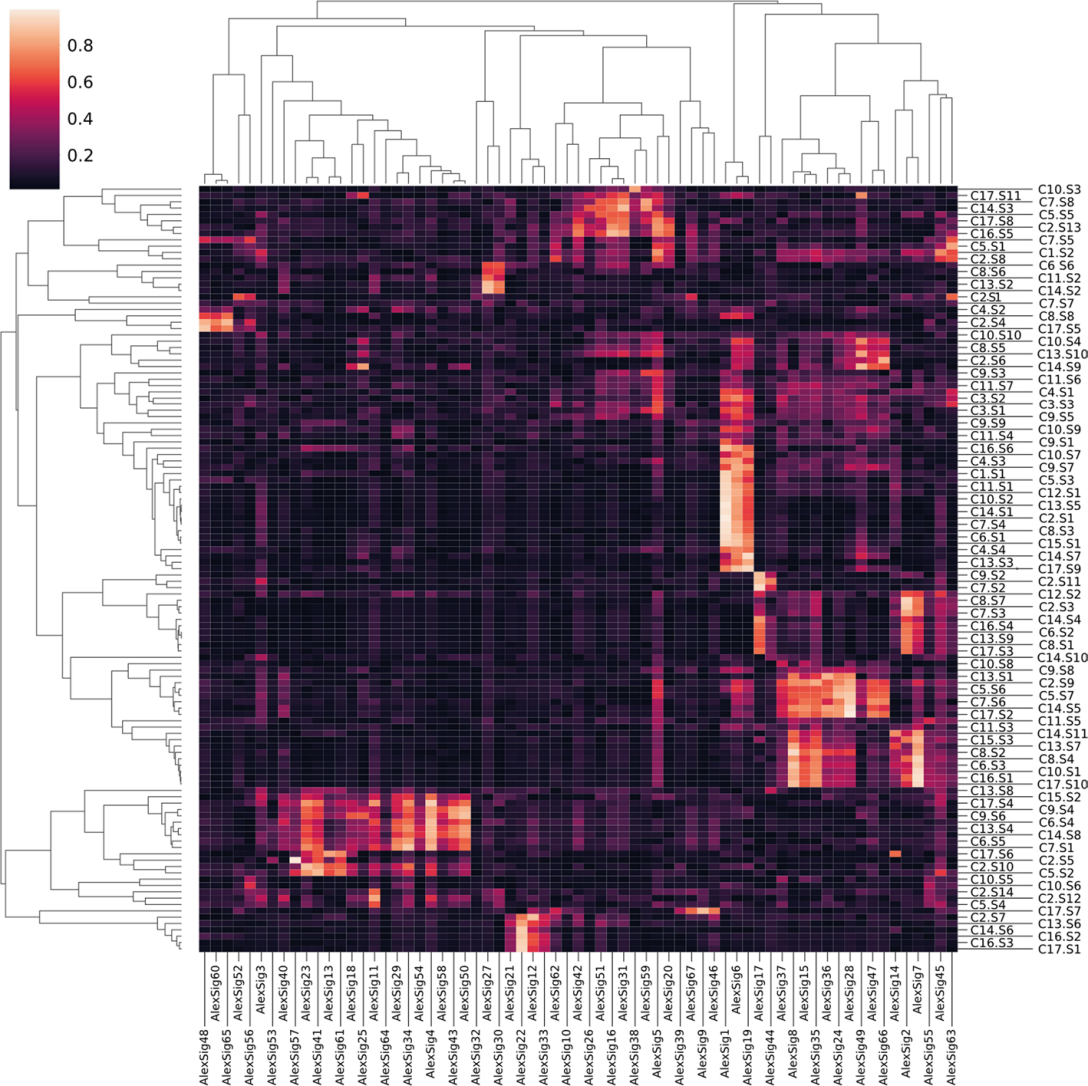
Cluster heatmap between our 121 signatures identified in our study and 67 COSMIC signatures. Ci.Sj shows identified signature ‘j’ from subtype ‘i’. In this figure, brighter cells correspond with a significant correlation and similarity

We also investigated mutational signatures in our identified subtypes. A mutational signature is a fingerprint for a molecular mechanism causing mutation across the genome. Molecular mechanisms are blind to what location they are causing the mutation. Therefore, to identify the molecular mechanism of the mutational signature, we have to consider all mutations in the whole genome (except mitochondria). We applied the CANCERSIGN tool [29] to complete mutational profiles of each subtype separately and identified 121 signatures. We then compared our signatures with 67 signatures identified in COSMIC [29]. We calculated the angular similarity between our identified signatures and COSMIC signatures to extract each signature’s biological information and their associated subtypes. A Heatmap of similarities between our signatures and Alexandrov signatures is shown in Fig. 5. Hierarchical clustering enables the finding of similar signatures beside each other.

As shown in the figure, COSMIC’s signature 1, whose number of mutations correlates with the individual’s age, is significantly correlated with many signatures in our identified subtypes, including C1.S1, C2.S1, and C12.S1. COSMIC’s signature 1 is shown to be highly associated with breast cancer. Interestingly, C1, C2, and C12 contain many breast cancer samples (27.4%, 16.8%, and 22%, respectively, as shown in Additional file 2: Figure S3). Also, COSMIC’s signature 2, attributed to the activity of the AID/APOBEC family of cytidine deaminases, is widely observed in the nervous system and is significantly correlated with signatures in C8 (C8.S7 and C8.S1), which consists of nervous system cancer (77.8%). Similarly, COSMIC’s signature 4 is also associated with smoking and is widely observed in lung cancer. It is highly correlated with two signatures in C6 (C6.S4, C6.S5), a subtype that consists of lung cancer patients (14.3%) and 20.4% of lung samples in C6. Similarly, COSMIC’s signature 5 is associated with skin cancer and is also correlated with a signature of C17 (C17.S8), which consists of skin cancer patients (38.5%).

Furthermore, COSMIC’s signature 6 is associated with defective DNA mismatch repair and is correlated with signatures from various subtypes, including C7.S4, C8.S3, C6.S1, and C15.S1. Also, COSMIC’s signatures 7 and 8 are related to ultraviolet light and skin cancer. These two signatures are highly correlated with signatures of C14 and C17 (C14.S11 and C17.S10), which consists of skin cancer (40.7% and 38.5%, respectively). Similarly, characteristics of COSMIC’s signature 16 are yet unknown but has observed in liver cancer tumors and are highly correlated with C7.S8 (9.9% of samples in this subtype are liver and 32.1% of liver samples are in C7) and C16.S5 (13.6% of samples in this subtype are liver). Also, COSMIC’s signature 22 is highly observed in Eso-AdenoCA cancer. This signature is correlated with C17.S1 (30.2% of samples in this subtype are Esophagus). Finally, COSMIC’s signature 34 is observed in samples from individuals with a tobacco chewing habit and has been found in oral and liver cancer. This signature is highly correlated with C6.S5 and C7.S1. 9.3% and 32.1% of liver samples are in C6 and C7, respectively.

The figure also shows that some signatures are presented in multiple subtypes. However, most of the signatures identified in each subtype are specific to a given subtype, indicating that samples within subtypes have the same mutational process. The exact amount of correlation between identified signatures and COSMICs (Alexandrov’s) and the correlation between each two identified signatures are provided in (Additional file 1: Table S7). The molecular mechanism respective to each mutational signature of COSMIC is also provided in this table.

The rate of different types of consequences of mutations caused by the molecular mechanisms is demonstrated in Additional file 2: Figure S6. As shown in this figure, not all subtypes have high impact consequences. However, as shown in multiple studies [30–32], even intronic mutations have an important role in cancer development.

### Gene ontology and pathway analysis

**Fig. 6:**
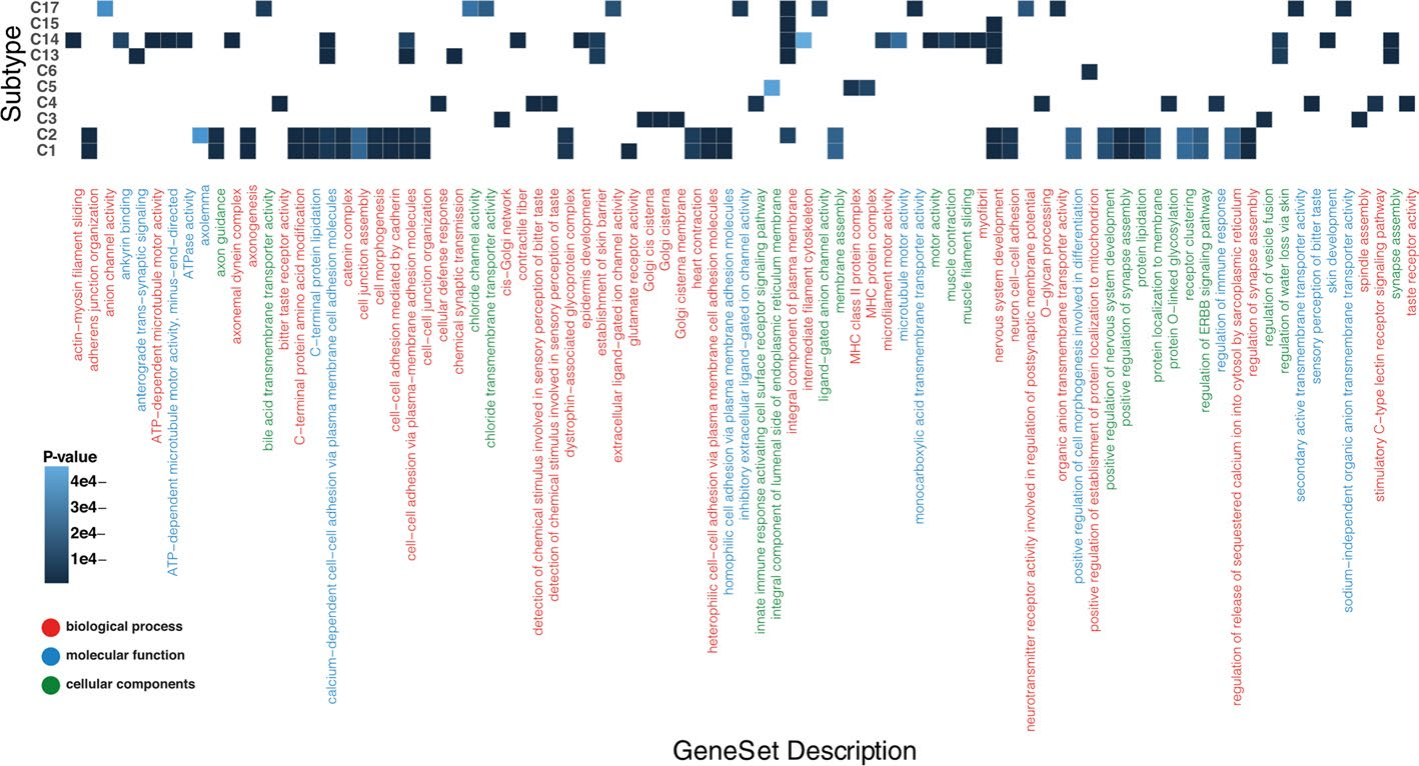
Gene ontology analysis of identified subtypes. For ten subtypes, we found enriched ontologies. The X-axis shows Gene-ontologies (a different color shows three collections of gene ontologies). The Y-axis shows subtypes, and the darkness of each cell corresponds to the *p*-value for enriched ontology. Many ontologies have significantly enriched for many subtypes, while there is a unique enriched gene ontology

We next investigate whether each subtype’s top 100 significant genes are associated with any gene ontology (GO) or gene pathway terms [33, 34]. We used the *enrichr* [35] package in R (*see method*) for gene ontology and pathway terms analyses. Gene ontology covers three main domains: biological process, molecular function, and cellular component. We considered all these domains and only retained enriched terms with FDR < 0.05. We identified at least one GO term for ten subtypes out of 17 (Fig. 6). Most GO terms are uniquely enriched in one subtype, while others are enriched in multiple subtypes.

For example, the “integral component of plasma membrane” is associated with five subtypes (C2, C13, C14, C15, and C17), and “nervous system development” is associated with another five subtypes (C1, C2, C13, C14, and C15). Conversely, the “bitter taste receptor activity”, “MHC class II protein complex”, “anterograde trans−synaptic signaling”, “actin−myosin filament sliding”, and “anion channel activity “ are examples of terms that are uniquely associated with C4, C5, C13, C14, and C17, respectively. Moreover, associated terms in C1 and C2 are almost the same, and only three terms associated uniquely in one of them (“axolemma” and “integral component of plasma membrane” are associated with C2 and “integral component of lumenal side of endoplasmic reticulum membrane” only associated with C1). Drugability and the complex effects of each element make it challenging to target pathways to restore the respective damaged functionality. However, each pathway can be targeted in multiple ways. Our findings can be a help in developing more precise drugs based on the subtype [36–38].

### Clinical report and survival analysis

**Fig. 7:**
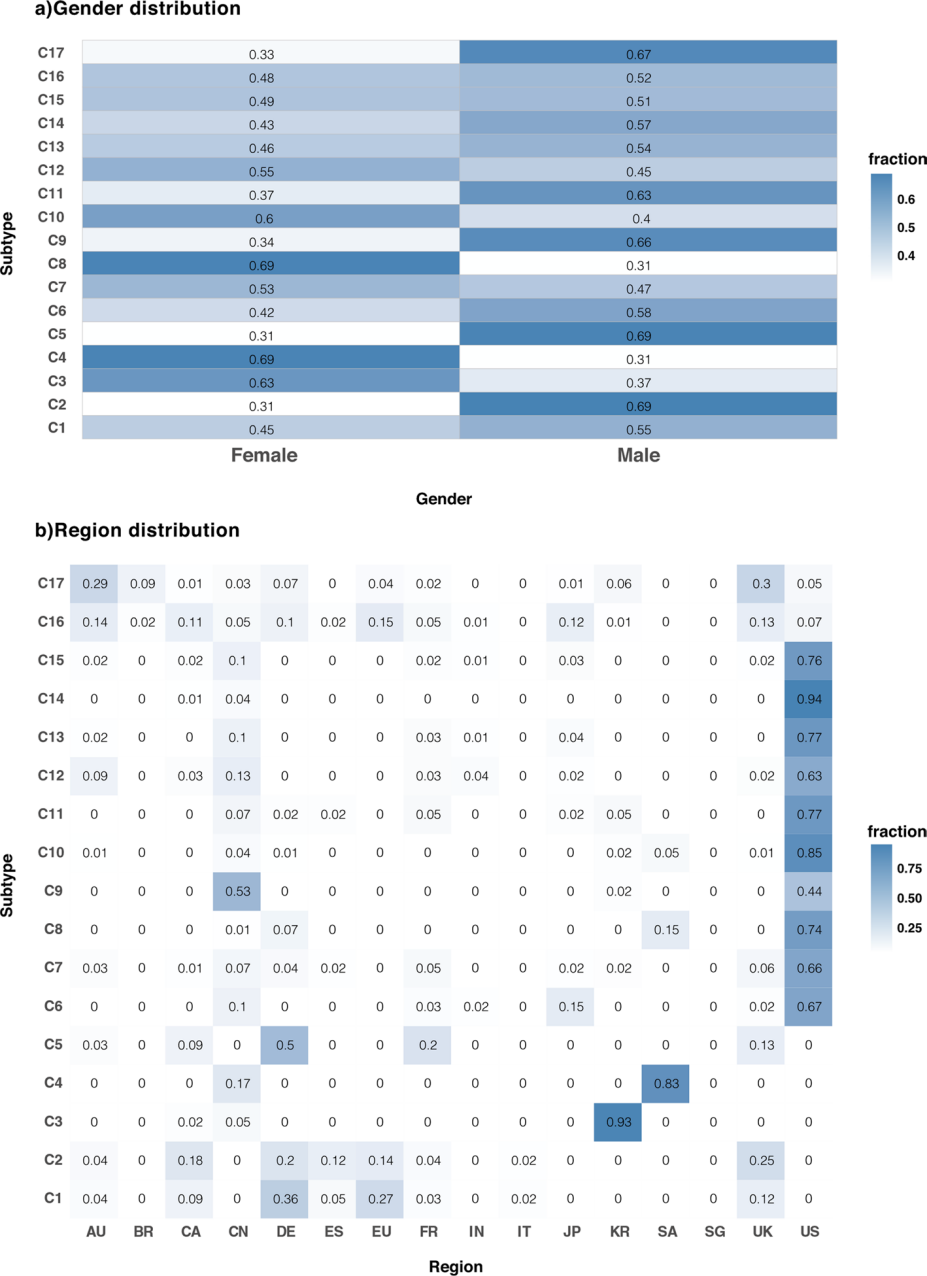
**a** Gender analysis of identified subtypes. The darkness of each cell corresponds to the fraction of male or female samples. Some subtypes (C3, C4, C8, and C10) are female-biased, while others, such as C2, C5, C9, C11, and C17, are male-biased. **b** Region distribution analysis of identified subtypes. The darkness of each cell corresponds to the fraction of samples that come from a specific region. Many subtypes are mainly from the US due to many samples from this country in the ICGC dataset, while C3 samples are mainly from Korea and C4 samples are mainly from Saudi Arabia

We also examined clinical data, such as gender and region (where the data were collected), available for a subset of the ICGC data. We identified several interesting results concerning the gender distribution of subtypes. For instance, C4 and C8, which mainly contain nervous system samples, are female-biased (69% of samples in these subtypes are female), and C2 (48.2% of samples are prostate cancer), C5 (84.3% of samples are prostate, blood, or brain cancers) and C17 (68.7% of samples are skin or esophagus cancers) are male-biased (Fig. 7a). The geographical distribution of identified subtypes is also shown in Fig. 7b.

**Fig. 8:**
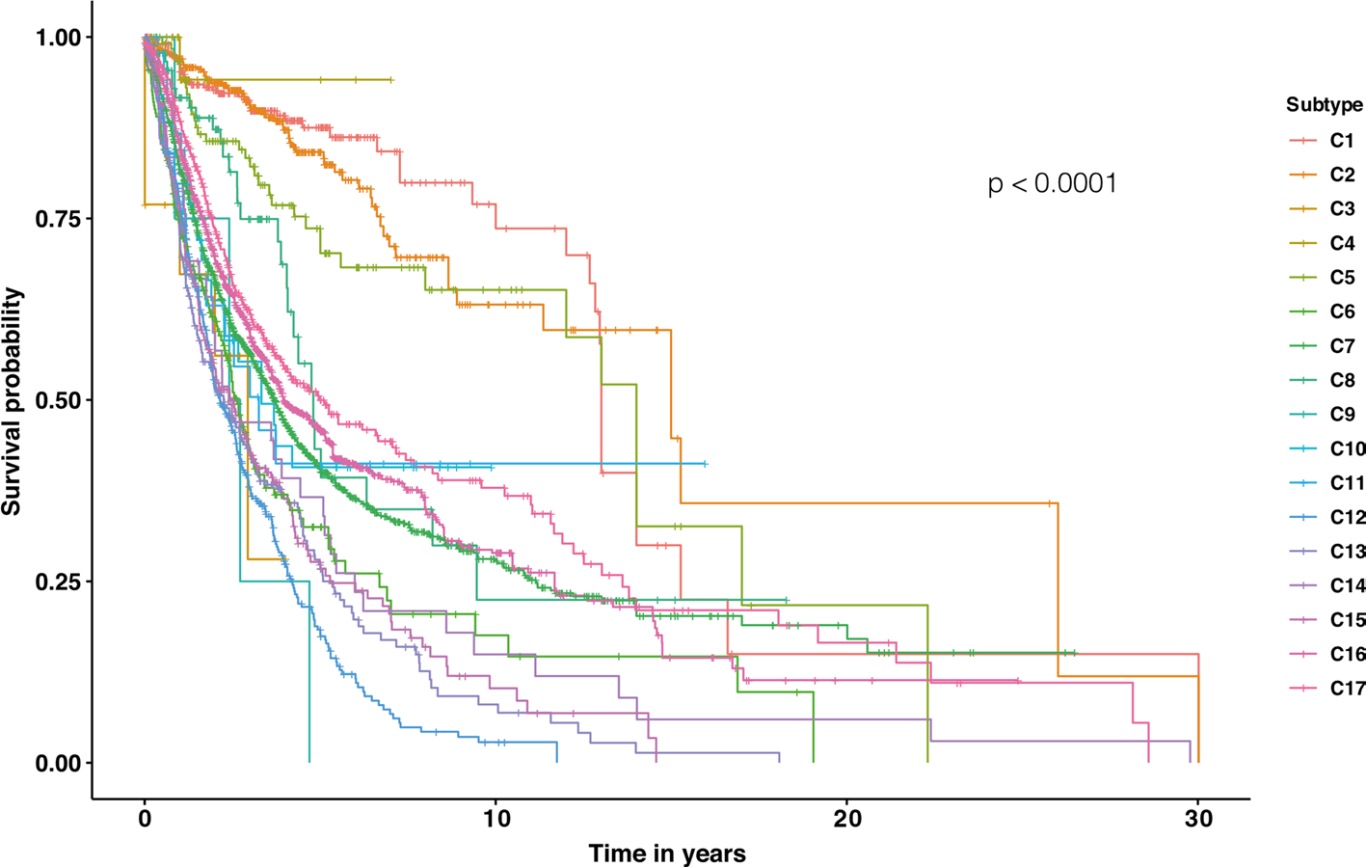
Survival analysis of identified subtypes. Each curve corresponds to a survival curve of a specific subtype. The X-axis shows time in years, and Y-axis shows a fraction of the survived samples. The survival curve demonstrates different survival times of different subtypes. Subtypes such as C3, C9, C12, and C15 have the worst survival, and samples in subtypes such as C1 and C2 have a higher chance of survival

We also used molecular data available for a subset of the ICGC dataset to investigate the difference between our identified subtypes regarding their survival curve. We begin by excluding samples of patients that were placed in different subtypes. We used the Kaplan–Meier [39] method to estimate survival probability over time and created survival curves for each subtype shown in Fig. 8. The *p*-value demonstrates the difference between subtypes. This figure shows that the survival times of identified subtypes are different. Since the data we use to cluster samples is entirely based on the somatic mutation data without any clinical information, this survival plot and *p*-value explicate influential biological signals.

As shown in Fig. 8, more than 75% of patients in C1 (significantly mutated in CSMD1/CNTNAP2) have a good survival length of 10 years. C2 and C5 (significantly mutated in DPP10/PTPRD and DMD, respectively) are also subtypes with a high chance of survival (survival of 13 years for more than 50% of their patients). However, patients in PCDHGA-driven subtype (C13), patients in PCDHA/PCDHGA-driven subtype (C14), and patients in PCDHA-driven subtype (C15) have the most unfortunate results since only 25% of patients of these subtypes have an overall survival of only five years. Moreover, NBPF/USP17-driven subtype (C3) and CSDE1/NRAS-driven subtype (C10) have the worst survival time (all patients in these two subtypes have a survival length of fewer than six years). Our results suggest that the Protocadherin family, USP17 family, NBPF family, NRAS, and CSDE1 substantially affect survival time.

## Conclusions

High-throughput sequencing has provided new opportunities to find the key mutations and molecular events by generating many samples. This led to accurate classification of patients based on their mutational profiles and, consequently, better clinical decisions on their treatment. This study used a new machine learning pipeline to propose a new clustering approach for cancer samples based on their mutational profiles. This can be useful in better understanding the underlying genetic causes of cancers by exploiting the context of the mutations in the driver genes in each subtype. We showed that considering both mutation rates in genes and the contexts of the mutations might be a more effective way to understand the molecular mechanism in cancer genomes. Our proposed pipeline helps discover mutational patterns associated with cancer-related pathways, clinical phenotypes, and cancer subtypes. The source codes for our proposed clustering pipeline and analysis are publicly available at: https://github.com/bcb-sut/Pan-Cancer.

## Materials and methods

This study performed a distribution-based analysis of genes and samples in which mutations occurred. We fitted the distribution for each cancer type and identified which genes are significantly mutated. We clustered all samples in all 19 cancer types and determined 17 cancer subtypes. Next, we comprehensively studied each subtype's phenotypic and genotypic characteristics to investigate differences and commonalities among different cancer subtypes. This includes: “Gene and Gene-motif association as a biomarker of each subtype”, “Mutational load of genes for each subtype”, “Mutational signature analysis”, “Gene ontology and pathway analysis” and “Clinical report and survival analysis”. Throughout this paper, the “cancer type” term indicates traditional cancer types identified by the tissue of origin and histopathology-based classification. At the same time, the “Cancer subtype” term indicates our newly proposed classes of cancers. In this section, we discuss our experiments and methods.

### ICGC dataset

We used the International Cancer Genome Consortium (ICGC) dataset, which contains data from 19 types of cancers. This dataset includes information about the sample’s location of mutation (based on comparing to the hg19 reference genome) such as chromosome, gene and allele number, type of mutation, and its consequences. In this study, we focus on somatic point mutations. We combined available data of each cancer type and then built the somatic mutation profile for 12,270 samples, of which 48.5% are female and 51.5% male. To determine which mutated genes were protein-coding, we used the FANTOMCAT database as a robust gene list (Accessed in May 2018) [40]. As a result, we identified 20,345 protein-coding regions mutated among all our samples. Then we annotated the genes with somatic mutation for all samples.

### Statistical pipeline to identify significant genes

Here we focused only on coding genes and identified significantly mutated ones in each cancer type in the following manner. We first counted the number of samples that had a mutation in each gene. We then used the Cullen-Frey graph [41] to find the best-fitted distribution for each cancer. Among different distributions, negative binomial demonstrated the best fit for our data. We have also experimentally investigated other distributions, and among them, the negative binomial distribution fitted the best to our data. We next used each cancer type’s best-fitted distribution (Fig. 1b) to identify significant genes. We then calculated the *p*-value for each gene in all cancers using the following formula:

1 $${\mathbf{P}}({\mathbf{x}} > {\mathbf{k}}) = 1 - \mathop {\mathop \sum \limits_{{{\mathbf{i}} = {\mathbf{r}}}} }\limits^{{\mathbf{k}}} {\mathbf{P}}\left( {{\mathbf{x}} = {\mathbf{i}}} \right) = 1 - \mathop {\mathop \sum \limits_{{{\mathbf{i}} = {\mathbf{r}}}} }\limits^{{\mathbf{k}}} \left( {\begin{array}{*{20}c} {{\mathbf{i}} - 1} \\ {{\mathbf{r}} - 1} \\ \end{array} } \right){\mathbf{p}}^{{\mathbf{r}}} {\mathbf{q}}^{{{\mathbf{k}} - {\mathbf{r}}}}$$

This is the probability of samples having more than k mutations in a given gene, where *p* is the probability that a sample has a mutation in a given gene (in this case, the relative frequency of mutated samples for each cancer type), and *q* is the complementary probability of having a mutation in a gene (not having a mutation in a given gene or *1 − p*). In the case of a gene mutating in 10 out of 100 samples of a given cancer type, *p* = *0.1* and *q* = *0.9*. This formula provides the *p*-value for determining whether a gene was significantly mutated in samples. The gene is considered to be significantly mutated if the *p*-value is less than the threshold.

Comparing the significance of obtained genes in each cancer is a challenging task. Still, if we select the mutated genes in a more significant portion of samples of each cancer type, we can get the genes primarily associated with cancer types. Therefore, genes located in the 0.001 right tail of the distribution (in other words, with a *p*-value less than 0.001) of each cancer type were selected to avoid unwanted redundancies. These 684 extracted genes are our features for the clustering step. For the rest of this paper, we refer to these genes as “Significant Genes.”

### Mutational load analysis

We performed mutational load analysis on protein-coding genes and the feature genes (candidate genes) for each subtype separately. Mutational load of gene ‘g’ in subtype ‘C’ is the number of samples in subtype C that mutated in gene g, divided by the total number of samples in subtype C.

### Mutational signature analysis

A mutational signature is a fingerprint for a molecular mechanism causing mutation across the genome. Molecular mechanisms are blind to what location they are causing the mutation. Therefore, to identify the molecular mechanism of the mutational signature, we have to consider all mutations in the whole genome (except mitochondria). We used CANCERSIGN to identify mutational signatures represented in our cancer samples [29]. Finding mutational signatures involves a Non-negative Matrix Factorization (NMF) computational method. Since this method is an unsupervised machine learning method (just like clustering) and the number of molecular mechanisms (hence mutational signatures) that are active among input samples is unknown, we have to run this algorithm multiple times to test multiple possibilities. Each time we assume that the number of signatures in the samples is *N*. We then change *N* each time in the range of 2–15. After calculating all the possibilities, results are tested in the form of evaluation plots provided in Additional file 2: Figure S1. We can decide which *N* is more accurate and optimal with the elbow rule. The complete procedures for selecting the optimal number of clusters are provided in the CANCERSIGN tool paper [23].

### Gene and gene-motif rates analyses

We used Fisher’s exact test to identify coding genes that significantly mutated in each subtype. Fisher’s exact test is done by computing a contingency table for each pair (gene, subtype). The contingency table consists of the number of samples in the subtype with a mutation in the gene, the number of samples in the subtype that had no mutation in the gene, the number of samples from other subtypes with a mutation in the gene, and the number of samples from different subtypes that had no mutation in the gene. We performed the same analysis for gene-motif to identify significantly mutated gene-motifs in each subtype. The results for the top 100 significant coding genes and top 100 significant gene-motifs for each subtype are shown in Additional file 1: Tables S4 and S5.

### Consequence type of mutations

The consequence type of mutations is available in the ICGC dataset. For each mutation, there may be multiple consequence types. We counted the consequences of each subtype’s significant genes and then calculated the frequency of consequence types for each subtype. Impact of consequence type of mutations was retrieved from here https://asia.ensembl.org/info/genome/variation/prediction/predicted_data.html in May 2022.

### Gene ontology analysis and gene pathway analysis on the significantly mutated coding genes

We used the gene ontology analysis tool enrichr [35] to observe the over-representation of gene ontology and pathways associated with each subtype’s top 100 significant genes separately. We used default value for adjusted *p*-value in enrichr (FDR < 0.05,). Gene ontology covers three domains: biological process, cellular component, and molecular function. The complete list of enriched gene ontology and pathway is provided in Additional file 1: Table S6.

### Clinical information

We downloaded clinical data for samples from ICGC (http://cancer.digitalslidearchive.net). Metadata files containing information about donors and their respective samples have been used to analyze gender and region. For each sample, we used the clinical data of the donor to whom the sample belonged. For gender analysis, we found the gender of each donor. But for ethnicity analysis, we used the project-code feature in ICGC metadata and extracted the region part from it to find the region where the sample was sequenced.

### Survival analysis

Like the Clinical report section, after obtaining the clinical data, specifically survival data, we filtered the patients so that all their samples belonged to a specific subtype. We used the Kaplan–Meier method to conduct survival curves for all subtypes. We used “survival”[42] and “survminer” [43] R packages to perform Kaplan–Meier curves and obtain the significance of survival prediction for subtypes. A Log-rank test was also applied to obtain the *p*-value for survival analysis.

## Supplementary Information

**Additional file 1**: **Table S1**. Complete list of candidate genes with their corresponding *P*-value for each cancer type separately. **Table S2**. Complete list of samples with their cancer type and identified subtypes. **Table S3**. Contribution of each cancer type in proposed cancer subtypes. **Table S4**. Top 100 significant genes with their corresponding *P*-value for each subtype separately. **Table S5**. Top 100 significant gene-motifs with their corresponding *P*-value for each subtype separately. **Table S6**: Full list of all enriched gene ontology associated with our identified subtypes. Sheet2: Full list of all enrich pathways associated with our identified subtypes. **Table S7**. Similarity of Cosmic Signatures and Pan-cancer subtypes.

**Additional file 2**: **Fig. S1**. Evaluation plots for finding optimal number of signatures. **Fig. S2**. Mutational load of feature genes in each cancer type. Fraction of samples that have mutated in each 684 candidate genes for all cancer types separately. The nervous system cancer type samples are less mutated. ALK and PTPN11 are the only significantly mutated genes in nervous system samples. Esophagus and skin cancer type have the most mutated samples. Different patterns of mutation are evident. The X-axis shows cancer types, and Y-axis shows the fraction of samples which had mutation in significant genes. **Fig. S3**. The fraction of different cancer types samples in each identified 17 subtypes is shown in the heat map. The X-axis shows identified subtypes, and Y-axis shows cancer types. Subtype C4 and C8 consist of head&neck samples primarily (82.8% and 77.8%, respectively). Prostate cancer is the most populated cancer in C1 and C2 (29% and 48.2% respectively), Skin cancer is the most populated cancer in C14 and C17(40.7% and 38.5% respectively), and Blood cancer is the most inhabited in C3 and C11 (68.1% and 37.2% respectively). **Fig. S4**. a) Mutational load of feature genes in C1 and C2 considering only samples with at least three mutations. Common highly mutated genes for both subtypes are shown. b) Mutational load of feature genes in C1 and C2 considering only samples with at least three mutations. Common highly mutated genes for both subtypes are shown. **Fig. S5**. Examle of motif rate in feature genes. a) Motif rate for IL1RAPL1 in C1, C2, and C5. b) Motif rate for IL1RAPL1 in C1, C2, C5, and C16. c) Motif rate for MUC16 in C4, C9, and C14. The X-axis shows 96 3-mer motifs, and Y-axis indicates the number of samples mutated in a specific motif divided by all samples. Each color corresponds to a particular class of 3-mer motifs. **Fig. S6**: Consequence type analysis. Rate of consequence type of mutations. The impact or severity of consequence of mutations are highlighted with different colors. Rate of high impact mutations are higher in some subtypes compared to others. For instance C9 has more high impact mutations among its samples compared to other samples. Some consequqnce types of mutations in ICGC dataset was not available in Ensembl database which is demonstrated as Unavailable impact.

## Data Availability

The source codes used in this Study for clustering and analysis of subtypes are provided in https://github.com/bcb-sut/Pan-Cancer.

## Declarations

### Experimental protocols

There are not any experimental protocols used in this study; all the data used in this study are publicly available. We also confirm that all statistics were carried out in accordance with relevant guidelines and regulations.
