# Supplementary material for: Pan-cancer integrative analysis of whole-genome De novo somatic point mutations reveals 17 cancer types
Source: BMC Bioinformatics. 2022 Jul 25;23:298. doi: 10.1186/s12859-022-04840-6 (PMC9316662; doi:10.1186/s12859-022-04840-6)
Supplement: Supplementary file 2 — Additional file 2: Fig. S1. Evaluation plots for finding optimal number of signatures. Fig. S2. Mutational load of feature genes in each cancer type. Fraction of samples that have mutated in each 684 candidate genes for all cancer types separately. The nervous system cancer type samples are less mutated. ALK and PTPN11 are the only significantly mutated genes in nervous system samples. Esophagus and skin cancer type have the most mutated samples. Different patterns of mutation are evident. The X-axis shows cancer types, and Y-axis shows the fraction of samples which had mutation in significant genes. Fig. S3. The fraction of different cancer types samples in each identified 17 subtypes is shown in the heat map. The X-axis shows identified subtypes, and Y-axis shows cancer types. Subtype C4 and C8 consist of head&neck samples primarily (82.8% and 77.8%, respectively). Prostate cancer is the most populated cancer in C1 and C2 (29% and 48.2% respectively), Skin cancer is the most populated cancer in C14 and C17(40.7% and 38.5% respectively), and Blood cancer is the most inhabited in C3 and C11 (68.1% and 37.2% respectively). Fig. S4. a) Mutational load of feature genes in C1 and C2 considering only samples with at least three mutations. Common highly mutated genes for both subtypes are shown. b) Mutational load of feature genes in C1 and C2 considering only samples with at least three mutations. Common highly mutated genes for both subtypes are shown. Fig. S5. Examle of motif rate in feature genes. a) Motif rate for IL1RAPL1 in C1, C2, and C5. b) Motif rate for IL1RAPL1 in C1, C2, C5, and C16. c) Motif rate for MUC16 in C4, C9, and C14. The X-axis shows 96 3-mer motifs, and Y-axis indicates the number of samples mutated in a specific motif divided by all samples. Each color corresponds to a particular class of 3-mer motifs. Fig. S6: Consequence type analysis. Rate of consequence type of mutations. The impact or severity of consequence of mutations are highlighted with [file 12859_2022_4840_MOESM2_ESM.pdf]

Supplementary figure S1. Evaluation plots for finding optimal number of signatures

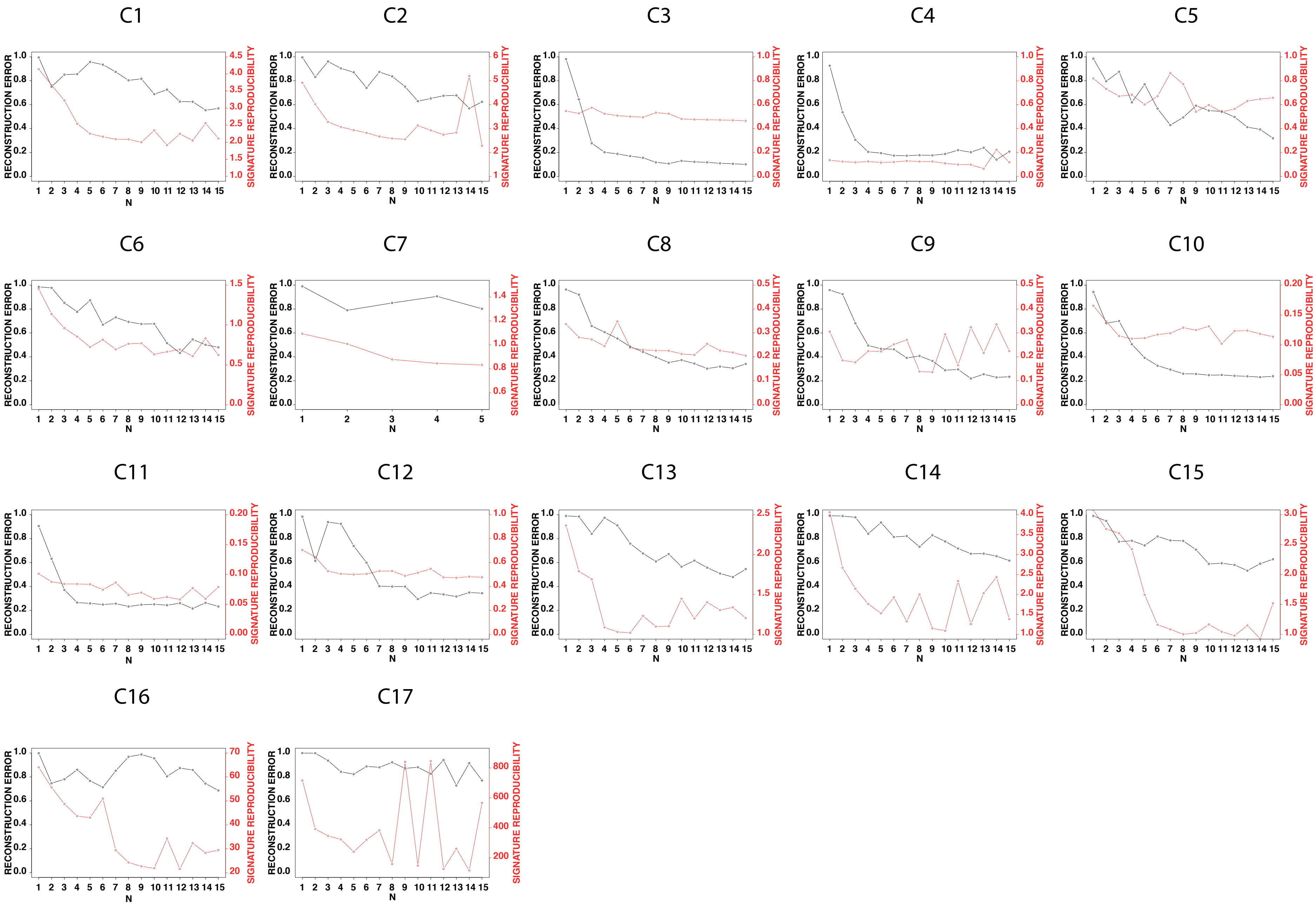

Supplementary figure S2. Mutational load of feature genes in each cancer type.

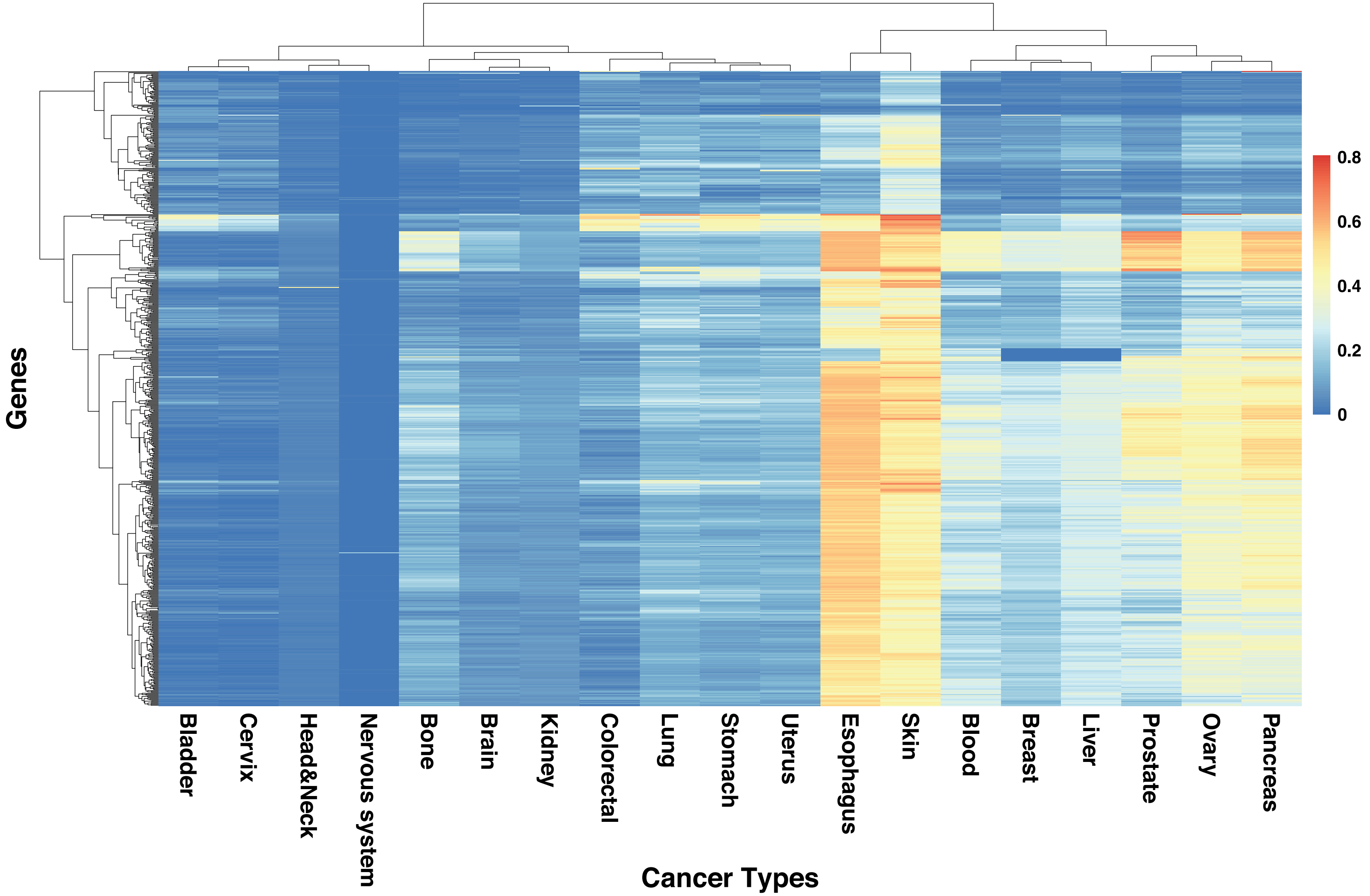

Supplementary figure S2. Mutational load of feature genes in each cancer type. Fraction of samples that have mutated in each 684 candidate genes for all cancer types separately. The nervous system cancer type samples are less mutated. ALK and PTPN11 are the only significantly mutated genes in nervous system samples. Esophagus and skin cancer type have the most mutated samples. Different patterns of mutation are evident. The X-axis shows cancer types, and Y-axis shows the fraction of samples which had mutation in significant genes.

Supplementary figure S3. Fraction of different cancer type samples in each 17 subtypes

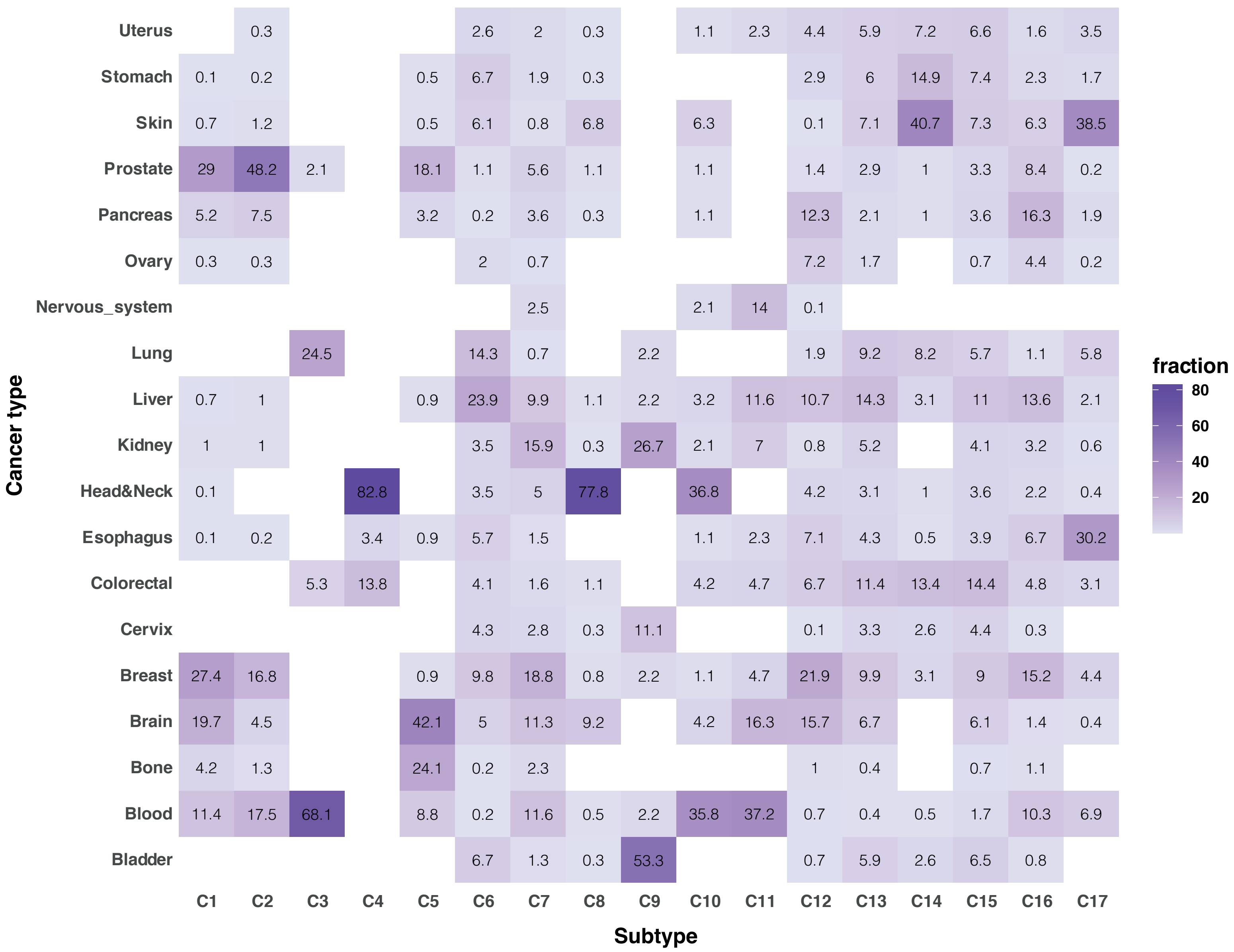

Supplementary figure S3. The fraction of different cancer types samples in each identified 17 subtypes is shown in the heat map. The X-axis shows identified subtypes, and Y-axis shows cancer types. Subtype C4 and C8 consist of head&neck samples primarily (82.8% and 77.8%, respectively). Prostate cancer is the most populated cancer in C1 and C2 (29% and 48.2% respectively), Skin cancer is the most populated cancer in C14 and C17(40.7% and 38.5% respectively), and Blood cancer is the most inhabited in C3 and C11 (68.1% and 37.2% respectively).

Supplementary figure S4. Mutational load of feature genes

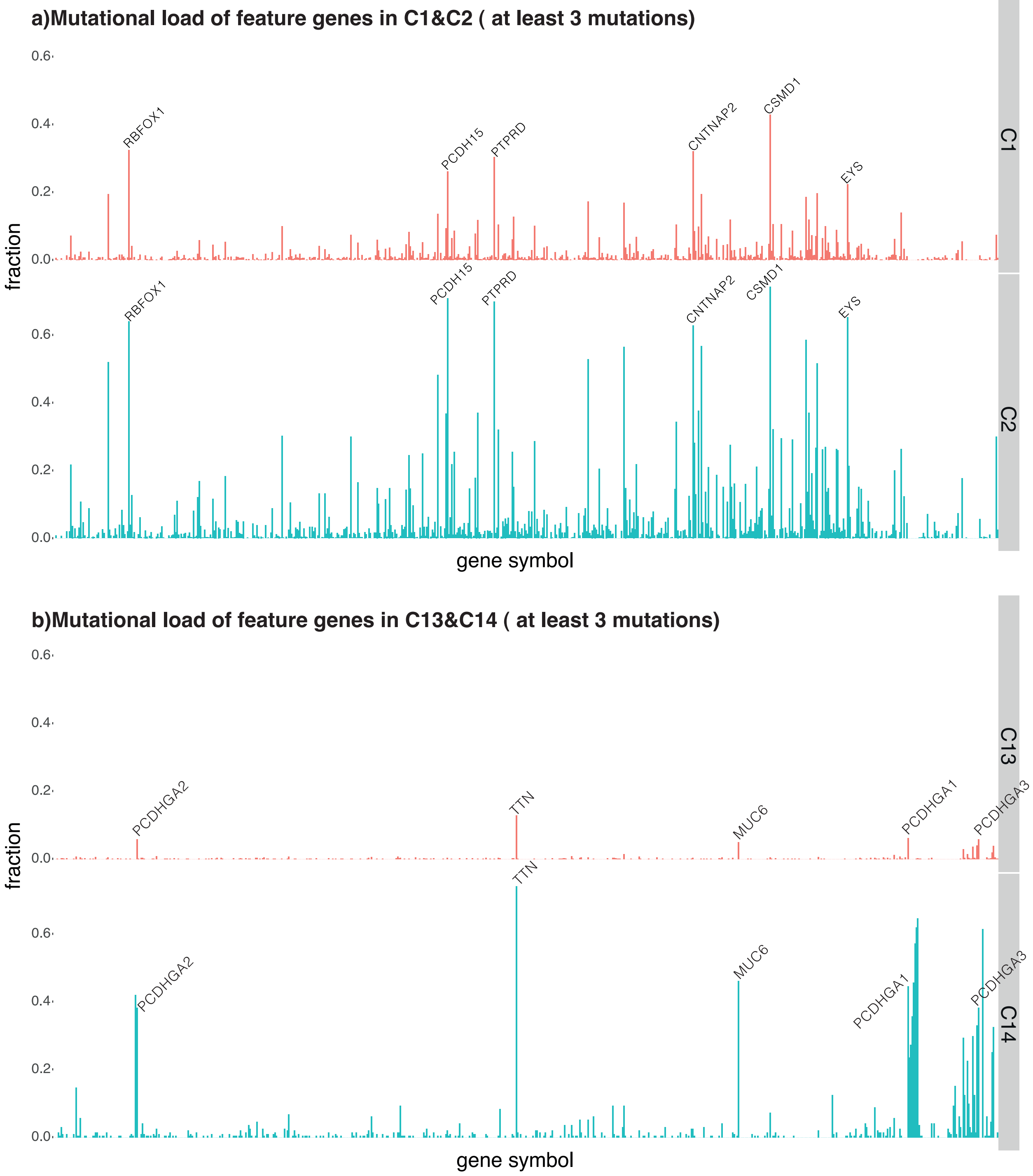

Supplementary figure S4. a) Mutational load of feature genes in C1 and C2 considering only samples with at least three mutations. Common highly mutated genes for both subtypes are shown. **b)** Mutational load of feature genes in C1 and C2 considering only samples with at least three mutations. Common highly mutated genes for both subtypes are shown.

Supplementary figure S5. examle of motif rate in feature genes

a)IL1RAPL1 gene

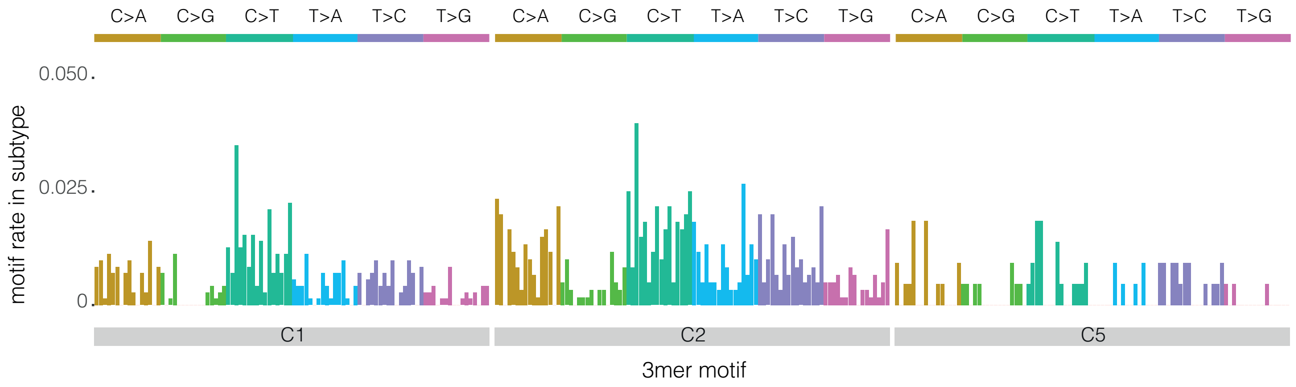

b)LRRC4C gene

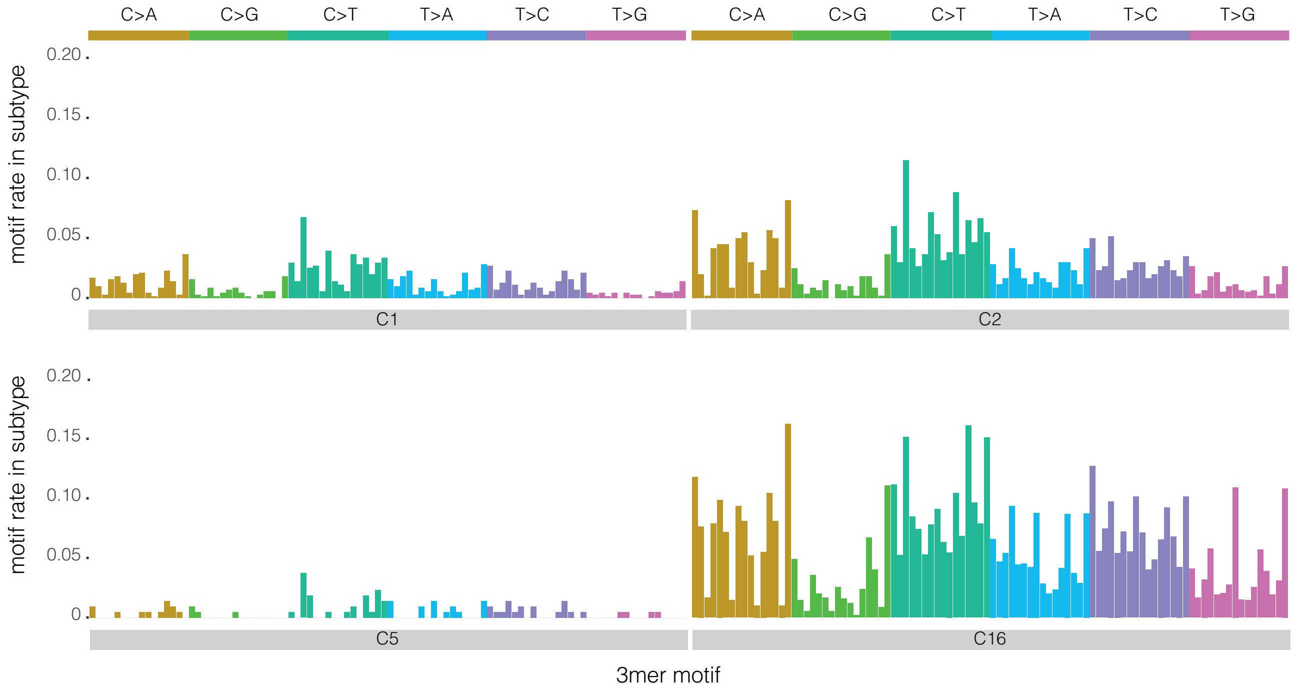

c)MUC16 gene

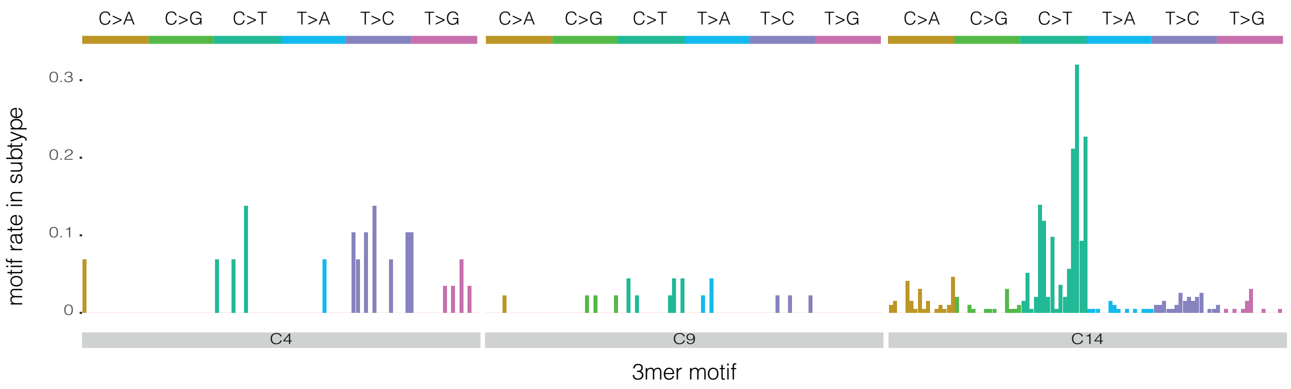

Supplementary figure S5. a) Motif rate for *IL1RAPL1* in C1, C2, and C5. **b)** Motif rate for *IL1RAPL1* in C1, C2, C5, and C16. **c)** Motif rate for *MUC16* in C4, C9, and

C14. The X-axis

shows 96 3-mer motifs, and Y-axis indicates the number of samples mutated in a specific motif divided by all samples. Each color corresponds to a particular class of 3-mer motifs.

Supplementary figure S6: Consequence type analysis

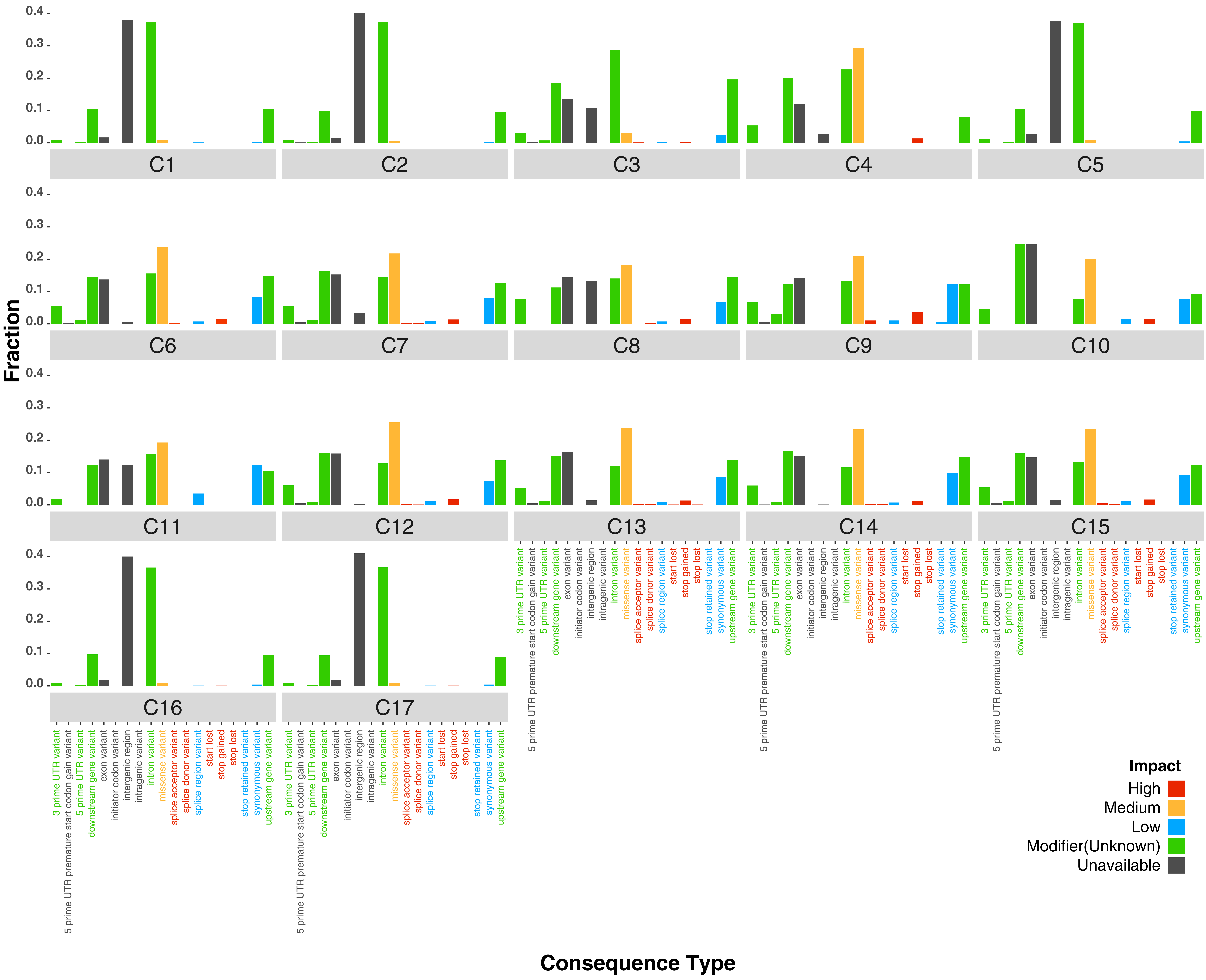

Supplementary figure S6. Rate of consequence type of mutations. The impact or severity of consequence of mutations are highlighted with different colors. Rate of high impact mutations are higher in some subtypes compared to others. For instance C9 has more high impact mutations among its samples compared to other samples. Some consequqnce types of mutations in ICGC dataset was not available in Ensembl database which is demonstrated as Unavailable impact.
